# Role of regulatory T cells in acute myeloid leukemia patients undergoing relapse-preventive immunotherapy

**DOI:** 10.1007/s00262-017-2040-9

**Published:** 2017-07-18

**Authors:** Frida Ewald Sander, Malin Nilsson, Anna Rydström, Johan Aurelius, Rebecca E. Riise, Charlotta Movitz, Elin Bernson, Roberta Kiffin, Anders Ståhlberg, Mats Brune, Robin Foà, Kristoffer Hellstrand, Fredrik B. Thorén, Anna Martner

**Affiliations:** 1 TIMM Laboratory, Sahlgrenska Cancer Center, University of Gothenburg, Medicinaregatan 1F, Box 425, 413 90 Gothenburg, Sweden; 2 Department of Hematology, University of Gothenburg, 413 45 Gothenburg, Sweden; 3 Department of Pathology and Genetics, Sahlgrenska Cancer Center, Institute of Biomedicine, Sahlgrenska Academy at University of Gothenburg, Medicinaregatan 1F, 413 90 Gothenburg, Sweden; 4 Department of Cellular Biotechnologies and Hematology, Sapienza University of Rome, Via Benevento 6, 00161 Rome, Italy

**Keywords:** Acute myeloid leukemia, Regulatory T cells, IL-2, Immunotherapy

## Abstract

Regulatory T cells (T_regs_) have been proposed to dampen functions of anti-neoplastic immune cells and thus promote cancer progression. In a phase IV trial (Re:Mission Trial, NCT01347996, http://www.clinicaltrials.gov) 84 patients (age 18–79) with acute myeloid leukemia (AML) in first complete remission (CR) received ten consecutive 3-week cycles of immunotherapy with histamine dihydrochloride (HDC) and low-dose interleukin-2 (IL-2) to prevent relapse of leukemia in the post-consolidation phase. This study aimed at defining the features, function and dynamics of Foxp3^+^CD25^high^CD4^+^ T_regs_ during immunotherapy and to determine the potential impact of T_regs_ on relapse risk and survival. We observed a pronounced increase in T_reg_ counts in peripheral blood during initial cycles of HDC/IL-2. The accumulating T_regs_ resembled thymic-derived natural T_regs_ (nT_regs_), showed augmented expression of CTLA-4 and suppressed the cell cycle proliferation of conventional T cells ex vivo. Relapse of AML was not prognosticated by T_reg_ counts at onset of treatment or after the first cycle of immunotherapy. However, the magnitude of T_reg_ induction was diminished in subsequent treatment cycles. Exploratory analyses implied that a reduced expansion of T_regs_ in later treatment cycles and a short T_reg_ telomere length were significantly associated with a favorable clinical outcome. Our results suggest that immunotherapy with HDC/IL-2 in AML entails induction of immunosuppressive T_regs_ that may be targeted for improved anti-leukemic efficiency.

**Electronic supplementary material:**

The online version of this article (doi:10.1007/s00262-017-2040-9) contains supplementary material, which is available to authorized users.

## Introduction

Regulatory T cells (T_regs_) are Foxp3^+^CD25^high^CD4^+^ T cells with diverse immunosuppressive functions. Subsets of T_regs_ include natural T_regs_ (nT_regs_), that are thymus-derived but undergo further expansion in peripheral tissues, and induced T_regs_ (iT_regs_) that are converted from conventional T cells (T_cons_) in the periphery [1–3]. Both subsets have been shown to suppress autoreactive lymphocytes and thus to limit the magnitude of innate and adaptive immune responses [2, 4, 5]. Accordingly, impaired T_reg_ function aggravates autoimmune diseases while T_reg_-mediated immunosuppression may inhibit pathogen clearance and promote chronic infection [6, 7]. In addition to controlling autoimmunity, T_regs_ have been ascribed a role as mediators of cancer-related immunosuppression. Studies in murine models show that T_regs_ accumulate in several forms of experimental cancer and that depletion of T_regs_ or strategies to target their immunosuppressive features reduce cancer growth [8, 9]. In many solid human cancers, T_regs_ accumulate in the tumor microenvironment and their presence typically, albeit not invariably, heralds advanced disease and poor survival [10–13].

Acute myeloid leukemia (AML) is characterized by rapid expansion of immature myeloid cells in bone marrow and other organs [14]. In AML, the malignant clone is reportedly controlled by cellular immunity, including natural killer (NK) cells and subsets of cytotoxic (CD8^+^) T cells [15]. While relatively little is known about the role of T_regs_ for the efficiency of anti-leukemic immunity in AML [16, 17], several other immunosuppressive pathways of relevance to the course of disease have been described [18–20] including immunosuppression exerted by NOX2-derived reactive oxygen species (ROS) released from myeloid cells [21]. Under conditions of NOX2-related oxidative stress, targeting of ROS formation using the NOX2 inhibitor histamine dihydrochloride (HDC) upholds NK cell and T cell function and improves the efficiency of NK- and T cell-activating agents such as interleukin-2 (IL-2) [22–25]. Monotherapy with IL-2 has yielded disappointment in several clinical trials in AML [26–31]. However, phase III trial results showed that the combination of HDC and low-dose IL-2 improves the leukemia-free survival (LFS) of AML patients in complete remission (CR) after chemotherapy [32], thus supporting the clinical relevance of NOX2-mediated immunosuppression in AML.

The IL-2 component of the HDC/IL-2 regimen may expand T_regs_ as these cells express high-affinity IL-2 receptors (CD25) and rely on exogenous IL-2 for proliferation [33, 34]. Treatment with IL-2 has been shown to increase the population of T_regs_ and reduce graft-versus-host manifestations in cancer patients receiving allogeneic stem cell transplants (allo-SCT) [35–38]. It is thus conceivable that IL-2-driven T_reg_ expansion may limit the anti-leukemic efficiency of HDC/IL-2 immunotherapy. For the present study, we monitored T_reg_ number and function in AML patients in first CR who received HDC/IL-2 for relapse prevention in a phase IV trial. Our results imply that treatment with HDC/IL-2 entails pronounced accumulation of nT_regs_ in blood and that aspects of T_reg_ function are relevant to relapse risk in AML.

## Patients, materials and methods

### Patients, study design and objectives

The Re:Mission trial (NCT01347996, registered at http://www.clinicaltrials.gov) was a single-armed multicenter phase IV study that enrolled 84 patients (age 18–79) with confirmed AML in first CR who were not eligible for allo-SCT. The patients received ten consecutive 21-day cycles of HDC/IL-2 during 18 months or until relapse or death. Each cycle comprised 0.5 mg histamine dihydrochloride (HDC; Ceplene^®^) and 16,400 U/kg human recombinant IL-2 (aldesleukin) that were administered by subcutaneous injection twice daily. The off-treatment periods in cycle 1–3 were 3 weeks, while the off-treatment periods between cycle 4–10 were extended to 6 weeks. All patients were followed for at least 24 months after enrollment. Fourteen patients discontinued prematurely from the study and were censored at the last captured follow-up date. The exclusion criteria for enrollment were identical to those employed in a previous phase III trial [32]. The primary endpoints comprised assessment of the quantitative and qualitative pharmacodynamic properties of HDC/IL-2, including monitoring of T and NK cell phenotypes before and after treatment cycles while analyses of aspects of immunity versus outcome (LFS and overall survival; OS) were performed post hoc. Patient characteristics, including details regarding previous induction and consolidation therapy and risk group distribution are accounted for in previous publications [39–41] and in Table 1. The trial was approved by the Ethical Committees of each participating institution and all patients gave written informed consent before enrollment.

**Table 1:** Patient characteristics

	*n* (%) All patients (*n* = 84)
Sex	
Female	44 (52)
Male	40 (48)
Risk group	
Favorable risk	34 (40)
Intermediate I	25 (30)
Intermediate II	13 (15)
High risk	7 (8)
Not done	5 (6)
Karyotype	
Normal	44 (52)
Favorable	14 (17)
Unfavorable	7 (8)
Other	15 (18)
Not done	4 (5)
Mutation status	
NPM1	*n* = 69 25 (36)
FLT3	*n* = 72 6 (8)
CEBPA	*n* = 42 3 (7)
Induction courses	
1	63 (75)
>1	21 (25)
Consolidation courses	
0–2	41 (49)
>2	43 (51)

### Isolation of PBMCs from healthy donors and patient samples

Buffy coats from healthy donors were obtained from the Blood Center at the Sahlgrenska University Hospital, Gothenburg, Sweden. To remove erythrocytes, the blood was mixed at a 1:1 ratio with 2% dextran and left to sediment. The upper phase was transferred to tubes containing Ficoll/Lymphoprep (Alere AB, Lidingö, Sweden) and peripheral blood mononuclear cells (PBMCs) were isolated by density gradient centrifugation. The PBMCs were cryopreserved until further use. Peripheral blood was collected from patients in the Re:Mission trial before and after the first and third treatment cycles, i.e., cycle 1, day 1 (C1D1) and cycle 1, day 21 (C1D21), cycle 3, day 1 (C3D1) and cycle 3, day 21 (C3D21). Patient PBMCs were isolated and cryopreserved at local sites and shipped on dry ice to the central laboratory (the TIMM Laboratory, Sahlgrenska Cancer Center, University of Gothenburg, Sweden) for analysis.

### Staining and flow cytometry

Cryopreserved samples were quickly thawed, washed and stained with LIVE/DEAD fixable yellow stain (Life technologies, Grand Island, NY, USA). Thereafter, cells were washed and incubated with an antibody cocktail for surface markers in PBS containing 0.5% BSA and 0.1% EDTA or in Brilliant stain buffer (BD Biosciences, Stockholm, Sweden). The following anti-human monoclonal antibodies were purchased from BD Biosciences: CD3-FITC (HIT3a), CD3-Brilliant Violet 711 (UCHT1), CD4-APC-H7 (RPA-T4), CD8-PerCP-Cy5.5 (SK1), CD14-FITC (MϕP9), CD25-Brilliant Violet 421 (M-A251), CD56-PE-Cy7 (NCAM16.2) and CD127-AF647 (HIL-7R-M21). CTLA-4-PE-Cy7 (L3D10) was obtained from Biolegend (San Diego, CA, USA) and CD14-Qdot655 (TüK4) from Life Technologies. For intracellular staining with Foxp3-PE (3G3; Miltenyi Biotec, Auburn, CA) and Helios-AF647 (22F6; BD Biosciences), cells were fixed and permeabilized using the Foxp3 fixation/permeabilization kit (eBioscience, San Diego, CA, USA) according to the manufacturer’s protocol. A 4-laser BD LSRFortessa SORP flow cytometer (405, 488, 532, and 640 nm; BD Biosciences) was employed to analyze samples. Data analysis was performed using the FlowJo software, version 7.6.5 or later (TreeStar, Ashland, OR, USA), or FACSDiva software, version 6 or later (BD Biosciences). Samples with less than 25% viability were excluded.

Blood samples were available from 81 out of 84 patients. Differential counts of whole blood were performed at local sites and were utilized to calculate absolute counts of T_regs_ in blood. Notably, the definition of T_regs_ in this study was restricted to Foxp3^+^CD25^high^CD4^+^ cells. All available samples were analyzed for T_reg_ content. If an analysis failed according to pre-defined criteria (experimental failure, few cells, poor cellular viability), a second sample was thawed for re-analysis. If the second attempt also failed to generate data, the sample was excluded from analysis. A thorough analysis of expression of T_reg_ markers (including CTLA-4 and Helios) was performed in 25 randomly selected patients. These patients were largely representative of all participating patients in terms of age (mean age for selected group 57.7 years (23.8–76.5 years) vs. mean age for all patients 58.6 years (19–77 years), risk group classification according to recommendations by the European LeukemiaNet [42] [among the selected patients 6 (24%) belonged to the favorable group, 14 (56%) to the intermediate group and 3 (12%) to the adverse group, 2 (8%) not done, whereas among all patients 34 (40.5%) belonged to the favorable group, 38 (45.2%) to the intermediate group and 7 (8.3%) to the adverse group, 5 (6%) not done] and French American British (FAB) classification (data not shown). All successfully analyzed samples, according to the pre-defined criteria stated above, were included in this report.

### T_reg_ methylation analysis

T_regs_ (CD4^+^CD14^−^CD25^hi^CD127^low^) were sorted from blood samples recovered at the end of treatment cycle 3 (C3D21) and from healthy subjects. Sorted cells (at least 40,000 cells per assay) were washed before being frozen in 200 µl PBS. The DNA methylation status of 15 CpG-motifs within the T_reg_-specific demethylated region (TSDR) was analyzed by bisulphite sequencing performed by Epiontis GmbH (Berlin, Germany) as previously described [43]. Only male subjects were included in analyses of T_reg_ methylation status cells since the *FOXP3* gene locus is located on the X-chromosome [44] and X-chromosome inactivation in females would likely influence results.

### T_reg_ suppression assay

Patient samples collected on C3D21 with a T_reg_ content of 15–40% of the CD4^+^ population were used in T_reg_ suppression assays ex vivo. PBMCs collected from healthy donors served as control. Cells were stained with anti-human monoclonal antibodies as described above. T_regs_ (CD4^+^CD14^−^CD25^hi^CD127^low^) and conventional CD4^+^ T cells (T_cons_; CD4^+^CD14^−^CD25^low^CD127^hi^) were sorted on a 3-laser BD FACSAriaIII flow cytometer (405, 488 and 640 nm; BD Biosciences). The gating strategy is shown in Supplementary Fig. 2. The sorted T_cons_ were stained with CellTrace™ violet (Life Technologies) and 35,000 cells per well were seeded together with 2 µg/ml soluble anti-CD28, in X-VIVO™ 15 serum-free medium (Lonza Group Ltd, Basel, Switzerland) to a 384-well plate coated with anti-CD3 (OKT3; eBioscience). An equal number of T_regs_ (35,000/well) was added to half of the wells. After 4–5 days of culture the proliferation of T_cons_ was determined by measuring the intensity of the CellTrace™ violet staining on an LSRFortessa SORP flow cytometer (BD Biosciences).

### Quantitative PCR telomere length assay

T_regs_ (CD4^+^CD25^hi^CD127^low^) were sorted from patient blood samples recovered at C3D1 and C3D21 or from healthy controls. Cells were sorted into 96-well plates (Life Technologies) for direct cell lysis and kept at −80 °C until analysis. Optimally, four technical replicates of 400 cells/well were obtained from all blood samples. Protease from *Streptomyces griseus* (2 μg; Sigma-Aldrich) diluted in PBS (Life Technologies) was added to each well followed by incubation at 37 °C for 10 min and enzyme inactivation at 95 °C for 15 min. The plates were centrifuged at 3000 rpm for 5 min. Quantitative PCR (qPCR) was performed using a CFX384 Touch Real-Time PCR Detection System (Bio-Rad). Primers designed by Cawthon [45] were used for amplification of a short fixed-length product at a copy number proportional to telomere length, and of the single copy gene albumin, in separate wells. Each 10-µl qPCR reaction contained 1X TATAA SYBR GrandMaster Mix (TATAA Biocenter), 400 nM of each primer, and 2 µl protease-treated DNA. Each technical replicate was assayed in duplicate. The thermal cycling profile was 95 °C for 1 min, 2 cycles of 94 °C for 15 s and 49 °C for 15 s, and 40 cycles of amplification (94 °C for 15 s, 62 °C for 10 s and 74 °C for 15 s). Formation of the correct PCR products was confirmed by melting-curve analysis. Relative telomere lengths were determined by normalizing the telomere qPCR signals against signals observed in the corresponding albumin gene assays.

### Statistical analyses

Single comparisons of T_reg_, T_con_ and NK cell phenotypes were performed by paired Student’s *t* test in accordance with the pre-defined statistical plan. Patients were dichotomized by the median T_reg_ cell number, frequency and telomere length for analyses of LFS (log-rank test). LFS was defined as the time in days from start of immunotherapy with HDC/IL-2 to relapse or death from any cause using data available at the trial closing date (October 13, 2014), i.e., when patients had been followed-up for at least 24 months. Cox multivariable regression analysis that included age and number of induction cycles as potential confounders was utilized to further determine the impact of T_reg_ distribution on LFS. Statistical analyses were performed using Graphpad Prism (Graph Pad Software, La Jolla, CA, USA) and IBM SPSS Statistics (IBM Corp., Armonk, NY, USA) software. All indicated *p* values are two-sided.

## Results

### Expansion of T_regs_ in blood during cycles of immunotherapy

**Fig. 1:**
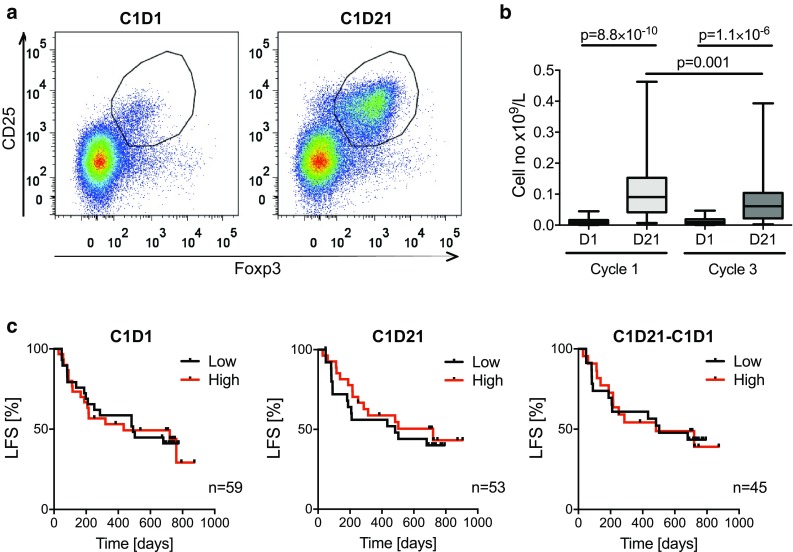
T_regs_ expand during immunotherapy with HDC/IL-2. **a** Representative dot plots of T_regs_ (defined as Foxp3^+^CD25^high^CD4^+^) before (cycle 1, day 1, C1D1) and after (C1D21) the first HDC/IL-2 treatment cycle. **b** Box plots represent blood counts of T_regs_ before (D1) and after (D21) cycles 1 and 3 of immunotherapy (C1D1 *n* = 59, C1D21 *n* = 53, C3D1 *n* = 51, C3D21 *n* = 50, Student’s paired *t* test). **c** Patients were dichotomized by the median for low number of T_regs_ in *black* and high number of T_regs_ in *red*, at onset of immunotherapy (C1D1; *left panel*) or end of cycle 1 (C1D21; mid panel). The right panel shows the LFS of patients with low or high induction of T_reg_ cell numbers during the first treatment cycle as analyzed by the log-rank test

Peripheral blood was drawn before and after the first and third 3-week cycle of HDC/IL-2 immunotherapy and analyzed for content of T_regs_ with Foxp3^+^CD25^high^CD4^+^ phenotype. A pronounced increase in the absolute numbers of blood T_regs_ (Fig. 1a, b) and in the percentage of T_regs_ among CD4^+^ cells (Fig. 4a) was observed during the first HDC/IL-2 treatment cycle. No significant changes in the absolute counts of Foxp3^−^CD4^+^ T cells were observed during treatment cycles (data not shown). T_reg_ levels in blood contracted to baseline levels after completion of a treatment cycle and were again induced during subsequent treatment cycles albeit to a significantly lower extent (Figs. 1b, 4a).

In a first attempt to determine the impact of T_reg_ levels on clinical outcome, patients were dichotomized by the median T_reg_ count at onset of therapy (cycle 1, day 1; C1D1) or after the first treatment cycle (C1D21) followed by analysis of LFS. The T_reg_ counts before or after the first treatment cycle did not predict LFS (Fig. 1c). Also, LFS did not differ between patients who were dichotomized based on high or low induction of T_regs_ during cycle 1 (Fig. 1c) or between patients in upper or lower quartiles of T_regs_ at onset or during therapy (*p* > 0.5, data not shown).

### The majority of expanded T_regs_ show stable Foxp3 expression

**Fig. 2:**
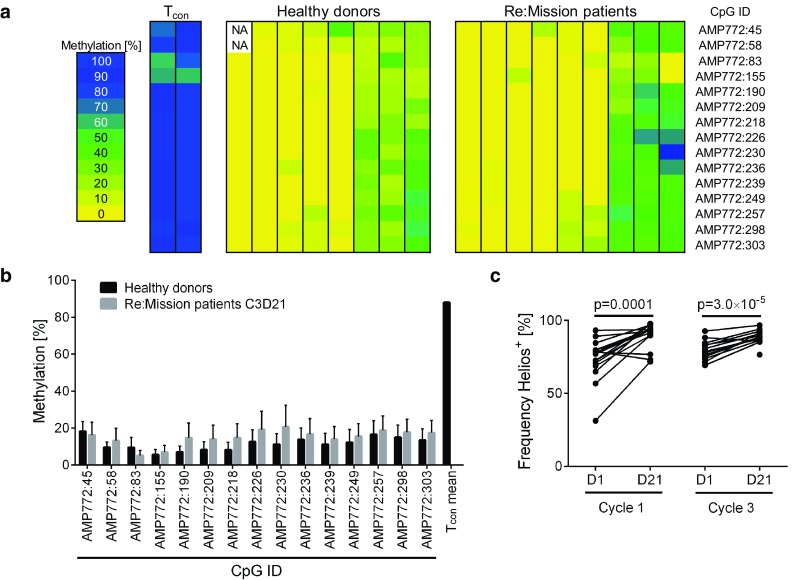
Expanded T_regs_ resemble thymic-derived nT_regs_. **a** Methylation pattern of 15 CpG islands in the TSDR, located in the *FOXP3* gene locus, for sorted T_cons_ from healthy donors (*n* = 2), sorted T_regs_ from healthy donors (*n* = 8) and sorted T_regs_ from Re:Mission patients (*n* = 9) with samples collected after treatment cycle 3 (C3D21). The *color code* indicates percentage methylation of each CpG island with *yellow* representing absence of methylation and blue 100% methylation. *NA* not analyzed. **b** *Bars* show the mean methylation of each CpG-site for healthy donors (*n* = 8) and Re:Mission trial patients (*n* = 9). *Error bars* display standard error of the mean (SEM). **c** Expression of Helios in T_regs_ before and after cycle 1 and cycle 3 of treatment with HDC/IL-2 (C1D1 *n* = 16, C1D21 *n* = 22, C3D1 *n* = 13, C3D21 *n* = 14). Statistical analyses were performed by Student’s paired *t* test

To determine the origin and stability of the expanded T_regs_, we analyzed the methylation status of the T_reg_-specific demethylated region (TSDR) in the *FOXP3* gene locus in T_regs_ purified after a HDC/IL-2 treatment cycle. A demethylated promoter reflects a stable Foxp3 expression, which is characteristic of thymic-derived nT_regs_. The TSDR region in T_cons_ as well as in iT_regs_ is, on the other hand, generally methylated [43, 46]. As shown in Fig. 2a, b, the TSDR in the *FOXP3* gene locus of the expanded T_regs_ was predominantly demethylated. The accumulating T_regs_ thus showed stable Foxp3 expression and hence resembled nT_regs_, which was further supported by their expression of Helios (Fig. 2c), a marker proposed to identify nT_regs_ [47].

### Immunosuppressive features of expanded T_regs_

**Fig. 3:**
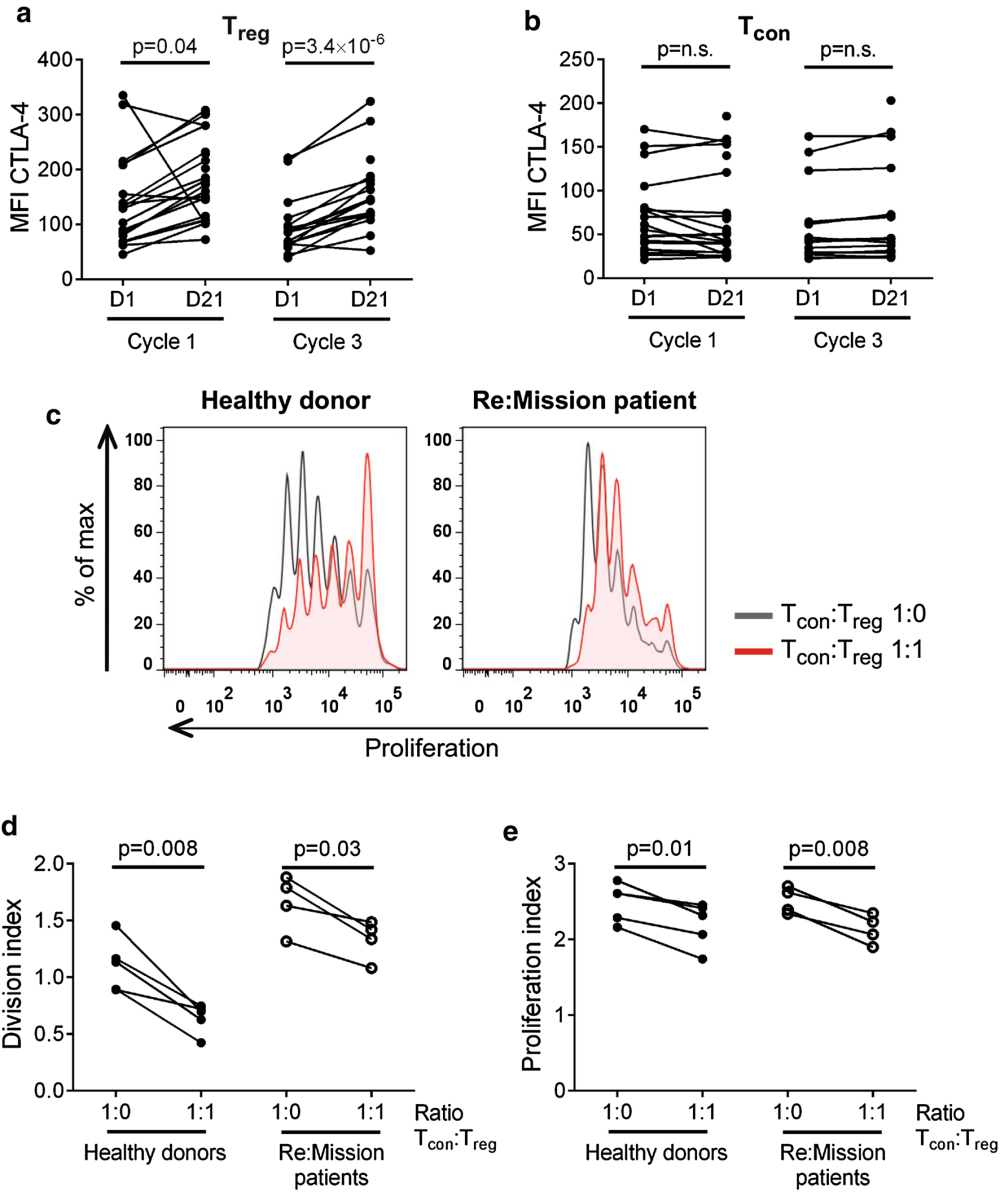
Expanded T_regs_ from Re:Mission trial patients are immunosuppressive. Median fluorescence intensity (MFI) of CTLA-4 on T_regs_ (**a**) and T_cons_ (**b**) in patient blood before and after treatment cycles 1 (C1D1 *n* = 19, C1D21 *n* = 25) and 3 (C3D1 *n* = 16, C3D21 *n* = 17). **c** Representative histograms of T_con_ proliferation from a healthy donor and a Re:Mission patient. *Black lines* show the proliferation of T_cons_ in wells without T_regs_ and *red shaded areas* show proliferation of T_cons_ when T_regs_ were added in a ratio of 1:1. Division index (**d**) and proliferation index **(e)** are shown for T_cons_ from healthy donors (*n* = 5) and Re:Mission trial patients (*n* = 4) at the end of treatment cycle 3. Statistical analyses were performed by Student’s paired *t* test

Numerous immunosuppressive features have been attributed to T_regs_, including the constitutive expression of the inhibitory receptor CTLA-4 [48]. During cycles of HDC/IL-2, the expression of cell surface CTLA-4 on T_regs_, but not on T_cons_, was significantly increased followed by contraction to baseline levels between cycles (Fig. 3a, b). In line with the above-referenced findings for T_reg_ induction (Fig. 1c), the expression level of CTLA-4 on T_regs_ did not significantly impact on patient outcome in terms of LFS (data not shown).

We next determined whether the accumulating T_regs_ retained the immunosuppressive features of normal T_regs_. To this end, T_regs_ (CD4^+^CD14^−^CD25^hi^CD127^low^) and T_cons_ (CD4^+^CD14^−^CD25^low^CD127^hi^) were FACS-sorted from patient blood after a treatment cycle followed by assessment of the proliferation of anti-CD3/anti-CD28-stimulated T_cons_ in the presence or absence of T_regs_. The patient-derived expanded T_regs_ reduced the proliferation of autologous T_cons_ as efficiently as did T_regs_ from healthy blood donors (Fig. 3c–e). Of note, the patient-derived T_cons_ proliferated more vigorously in response to anti-CD3/anti-CD28-stimulation compared with healthy donor T_cons_ (Fig. 3c, d), likely reflecting their primed status at the end of a HDC/IL-2 cycle.

### T_reg_ exhaustion and short T_reg_ telomere length predict favorable clinical outcome

**Fig. 4:**
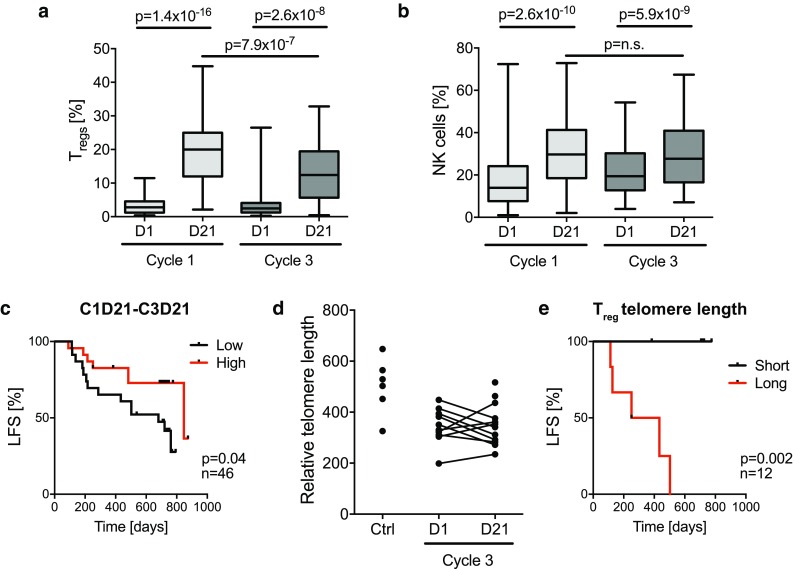
Expansion of T_regs_ is reduced in later cycles of immunotherapy. Box plots display (**a**) the frequency of T_regs_ within the CD4^+^ compartment (C1D1 *n* = 59, C1D21 *n* = 63, C3D1 *n* = 52, C3D21 *n* = 53), and (**b**) frequency of NK cells as percentage of lymphocytes (C1D1 *n* = 62, C1D21 *n* = 63, C3D1 *n* = 53, C3D21 *n* = 53), before (D1) and after (D21) the first and third HDC/IL-2 treatment cycle. Statistical analyses were performed by Student’s paired *t* test. **c** Patients were dichotomized by the median for low (*black*) or high (*red*) reduction in T_reg_ percentage from the end of cycle 1 to the end of cycle 3, and LFS was analyzed by the log-rank test. **d** Relative telomere length of T_regs_ FACS-sorted from patient blood obtained before and after the third treatment cycle or from healthy blood donors (Ctrl). **e** Kaplan–Meier plot comparing the LFS of patients with T_reg_ telomere lengths on C3D21 below (*black*) and above (*red*) the median (log-rank test)

The analyses accounted for above indicated that the T_regs_ that accumulated during HDC/IL-2 immunotherapy did not negatively impact on clinical outcome despite showing features of immunosuppression. In addition to T_regs_, NK cell counts were markedly increased in blood during treatment cycles of HDC/IL-2 (Fig. 4a, b). The favorable impact of aspects of NK cell biology on the outcome of patients in this trial is described in detail elsewhere [40, 41]. To further elucidate the reasons for the apparent inability of the accumulating T_regs_ to adversely affect patient outcome, we compared the kinetics of T_reg_ and NK cell accumulation during immunotherapy. As shown in Fig. 4a, b, the magnitude of T_reg_ induction, but not that of NK cell induction, was reduced in later treatment cycles. Furthermore, patients displaying high reduction in the fraction of T_regs_ at the end of cycle 3 compared with the end of cycle 1 showed significantly improved LFS (Fig. 4c). This difference remained significant in a multivariable analysis correcting for potential confounders for LFS (p = 0.025, Cox multivariable regression analysis).

To clarify the mechanism underlying the decline in T_reg_ induction during later treatment cycles, we set up an assay to determine telomere length by qPCR. T_regs_ were FACS-sorted from patient samples before and after treatment cycle 3 and analyzed for telomere length. The T_reg_ telomere length did not differ significantly before and after a treatment cycle (Fig. 4d). However, short T_reg_ telomeres at the end of treatment cycle 3 were significantly associated with reduced relapse risk (Fig. 4e).

## Discussion

Upon diagnosis, AML patients receive induction chemotherapy aiming to achieve CR, which is defined as the microscopic disappearance of leukemic cells and the return of normal hematopoiesis. Despite additional courses of chemotherapy (consolidation), relapse in CR is common and significantly explains why the long-term survival of adult AML patients remains in the range of 30–40% [14]. A large body of evidence, including the graft-versus-leukemia reaction that mediates relapse prevention after allo-SCT, implicates functions of cytotoxic T cells and NK cells in controlling the malignant clone in AML [15, 39–41]. The purported role of cell-mediated immunity for the surveillance of leukemic cells in AML has inspired the development of immunotherapeutic strategies, in particular for patients in CR who harbor a minimal yet potentially life-threatening burden of leukemia (reviewed in [15]).

HDC/IL-2 is currently the only documented effective non-transplant immunotherapy for relapse prevention in AML beyond the chemotherapy phase [15, 32]. As the IL-2 component of this regimen may induce T_regs_ [35–38] the present study was designed to determine the magnitude of T_reg_ induction during immunotherapy, the origin and function of accumulating T_regs_ and the potential impact of T_regs_ on relapse risk. We therefore analyzed serial blood samples from patients in first CR participating in the phase IV Re:Mission trial (*n* = 84) who received ten 3-week cycles of HDC/IL-2 after the completion of consolidation chemotherapy. The frequency of T_regs_ at the onset of immunotherapy was within or below the range in healthy subjects (3.1 ± 2.2% of CD4^+^ T cells; mean ± SD), which is in agreement with a recent study of AML patients in CR [49]. T_reg_ counts increased considerably during cycles of HDC/IL-2, in particular during the first treatment cycle. At the end of the first cycle, T_regs_ typically comprised 15–25% of the CD4^+^ cell population in blood. These results concur with previous reports of T_reg_ induction during treatment of cancer patients with IL-2 [35, 37, 38] and is likely explained by IL-2 acting via the high-affinity IL-2 receptor CD25 that is constitutively expressed by nT_regs_. However, randomized comparisons are required to exclude the possibility that the HDC component contributed to T_reg_ induction. While we did not have access to bone marrow samples in this study, we reason that a similar increase in T_reg_ counts is likely to occur also in the bone marrow, since the number of T_regs_ in blood and bone marrow were previously reported to be highly correlated [50].

We then asked whether the expanded population of T_regs_ showed stable or transient expression of Foxp3. In these cells, the TSDR in the *FOXP3* gene locus was highly demethylated implying stable Foxp3 expression and suggesting that the reduction of T_reg_ counts between cycles was explained by T_reg_ apoptosis rather than the T_regs_ being reprogrammed into T_cons_. Moreover, there was no increase in the number of T_cons_ during or between treatment cycles (data not shown). The thymus-derived nT_regs_ are known to have a demethylated TSDR in the *FOXP3* gene locus while this region generally is more methylated in iT_regs_. With the precaution that the TSDR region may become demethylated also in iT_regs_ in response to antigen stimulation in the presence of IL-2 [51], we propose that the expanded T_regs_ were mainly derived from proliferating nT_regs_.

We observed that the T_regs_ accumulating at the end of a HDC/IL-2 treatment cycle expressed elevated levels of CTLA-4, which reportedly contributes to the immunosuppression exerted by these cells [48]. Also, the expanded T_regs_ suppressed the proliferation of T_cons_ in co-culture assays ex vivo. While it is conceivable that T_reg_ induction may dampen the development of cell-mediated immunity of relevance to elimination of residual leukemia, our initial analysis did not reveal associations between the magnitude of T_reg_ induction during initial cycles of immunotherapy and clinical outcome. It is conceivable, however, that the lack of association between T_reg_ induction and clinical outcome may result from effects of HDC—a NOX2 inhibitor—on the immunosuppressive properties of T_regs_. This possibility is supported by a previous study showing that immunosuppressive features of CD8^+^ T_regs_ rely on functional NOX2 [52]. However, monotherapy with IL-2 has been reported to increase T_reg_ counts and limit the extent of graft-versus-host disease (GvHD) after allo-SCT in cancer patients, apparently without negatively affecting survival [35]. In accordance, results presented by Martelli et al. implied that allo-transplanted patients with acute leukemia who received donor-derived T_regs_ in conjunction with T_cons_ for protection against GvHD did not show increased relapse risk [53].

A more detailed analysis of T_reg_ kinetics during treatment with HDC/IL-2 revealed that aspects of T_reg_ function may indeed impact on clinical outcome. We observed that the magnitude of T_reg_ induction was frequently blunted in later treatment cycles and that a reduced T_reg_ accumulation in cycle 3 weakly but significantly prognosticated low relapse risk, thus supporting that sustained presence of T_regs_ may adversely impact on prognosis. In contrast, the induction of NK cells in blood remained largely stable throughout cycles of immunotherapy. The mechanisms underlying the different kinetics of NK cell and T_reg_ induction should be further studied. However, in people over the age of 45 the supply of thymic nT_regs_ is minimal and is sustained mainly by peripheral proliferation [54]. We thus speculate that the supply of nT_regs_ may become exhausted during repeated cycles of immunotherapy, in contrast to the bone marrow supply of NK cells. In support of this assumption, we observed a significantly reduced accumulation of T_regs_ in later treatment cycle only in patients  >45-years-old (Supplementary Fig. 1a).

The proliferation of normal somatic cells is limited by the length of telomeres, which typically progressively shorten with increasing age [55]. Accordingly, we observed a significant correlation between short T_reg_ telomere length and age among the participating patients (Supplementary Fig. 1b). Despite high age being a dominant predictor of relapse risk in AML [56], short T_reg_ telomeres at the end of a treatment cycle was observed mainly in older patients and significantly prognosticated favorable LFS. In agreement with the above-referenced hypothesis of T_reg_ exhaustion during immunotherapy, we propose that short T_reg_ telomere length may reflect a reduced capacity of nT_regs_ to undergo proliferation and, hence, exert immunosuppression in subsequent treatment cycles.

While the preliminary nature of these findings should be emphasized, we speculate that immunosuppressive nT_regs_ may be targeted for improved anti-leukemic efficacy of HDC/IL-2 immunotherapy. This view gains support from previous studies in which T_regs_ were targeted during immunostimulation with IL-2 in experimental leukemia using the combination of anti-CD25, aiming to deplete T_regs_, and IL-2. This combination significantly improved the survival of leukemia-bearing mice over either treatment alone [57]. In further support for a role of T_regs_ in AML immunotherapy, Bachanova et al. reported that patients with relapsed or refractory AML showed encouraging CR rates and disease-free survival following depletion of host T_regs_ prior to the adoptive transfer of haploidentical NK cells and IL-2 [58]. Targeting T_regs_, for example by use of antibodies blocking CTLA-4, may thus be considered in IL-2-based AML immunotherapy. An alternative approach to minimize a potential negative impact of T_regs_ may be to replace the IL-2 component with modified IL-2 variants or IL-15 that activate anti-leukemic effector cell populations with reduced or absent expansion of CD25^high^ expressing T_regs_ [59–61].

## Electronic supplementary material

Below is the link to the electronic supplementary material.

Supplementary material 1 (PDF 88 kb)

## Abbreviations

Allo-SCT: Allogeneic stem cell transplant
AML: Acute myeloid leukemia
C1D1: Cycle 1, day 1
C1D21: Cycle 1, day 21
C3D1: Cycle 3, day 1
C3D21: Cycle 3, day 21
CR: Complete remission
GvHD: Graft-versus-host disease
HDC: Histamine dihydrochloride
iT_regs_: Induced regulatory T cells
LFS: Leukemia-free survival
NOX2: Nicotinamide adenine dinucleotide phosphate oxidase isoform 2
nT_regs_: Natural regulatory T cells
OS: Overall survival
qPCR: Quantitative PCR
ROS: Reactive oxygen species
T_cons_: Conventional T cells
T_regs_: Regulatory T cells
TSDR: Regulatory T cell-specific demethylated region
