# Supplementary material for: Role of regulatory T cells in acute myeloid leukemia patients undergoing relapse-preventive immunotherapy
Source: Cancer Immunol Immunother. 2017 Jul 18;66(11):1473–84. doi: 10.1007/s00262-017-2040-9 (PMC5645432; doi:10.1007/s00262-017-2040-9)
Supplement: Supplementary file 1 — Supplementary material 1 (PDF 88 kb) [file 262_2017_2040_MOESM1_ESM.pdf]

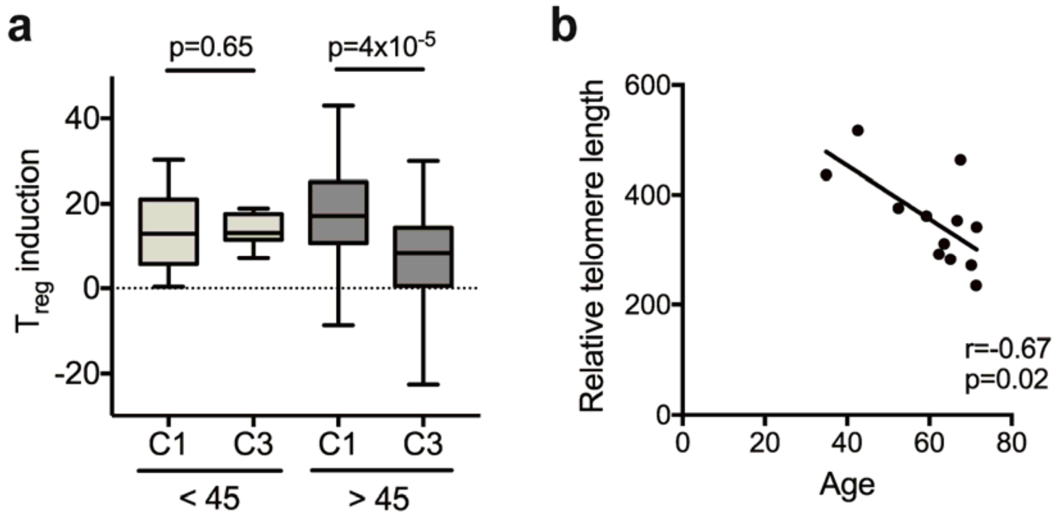

Supplementary figure 1. Older patients have shorter T_{reg} telomeres and reduced capacity to expand T_{regs} in later HDC/IL-2 treatment cycles. (a) Induction of T_{regs} (percentage out of $CD4^+$ cells) during cycle 1 (C1; C1D21-C1D1) and cycle 3 (C3; C3D21-C3D1) of HDC/IL-2 immunotherapy in trial patients below and above 45 years of age (< 45: C1 n=9, C3 n=9; >45: C1 n=44, C3 n=39). The induction of T_{regs} during the two cycles was compared using Student´s paired t-test. **(b)** Illustration of the correlation (Pearson's *r*) between relative T_{reg} telomere length on C3D21 and age of the assayed patients (n=12).

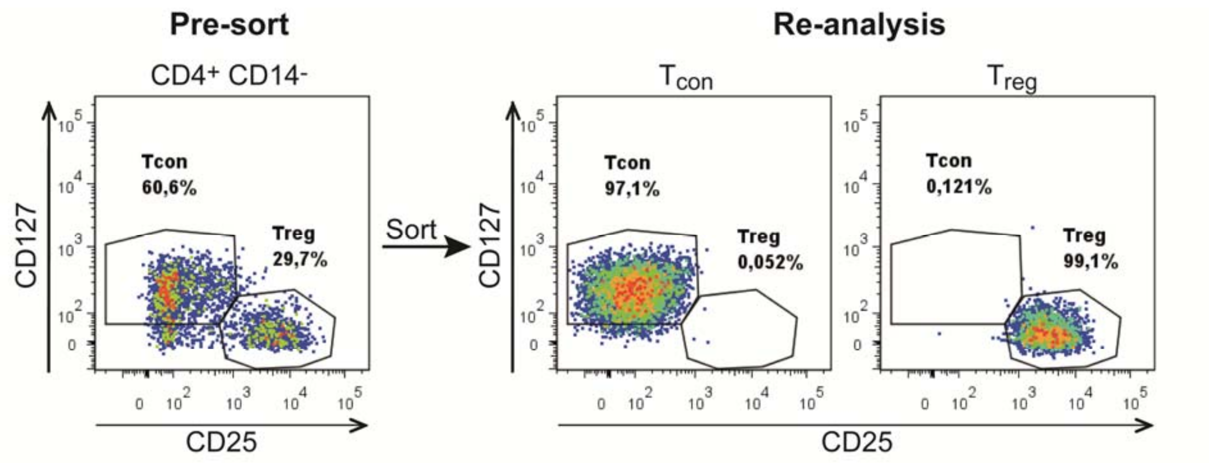

Supplementary figure 2. Gating strategy for sort of T_{regs} and T_{cons}. $CD4^{+}CD14^{-}$ cells were further gated on CD25 and CD127 to distinguish T_{cons} ($CD25^{low}CD127^{high}$) from T_{regs} ($CD25^{high}CD127^{low}$). The left dot plot shows a sample before sorting. The purity of sorted populations was determined by re-analysis of sorted samples (dot plots to the right).
